# Effects of Hinged versus Floor-Reaction Ankle-Foot Orthoses on Standing Stability and Sit-to-Stand Performance in Children with Spastic Diplegic Cerebral Palsy

**DOI:** 10.3390/ijerph19010542

**Published:** 2022-01-04

**Authors:** Yu-Lin Wang, Wen-Chou Chi, Chiung-Ling Chen, Cheng-Hsieh Yang, Ya-Ling Teng, Kwok-Tak Yeung

**Affiliations:** 1 Center for General Education, Southern Taiwan University of Science and Technology, Tainan 710301, Taiwan; d8101080@gmail.com; 2 College of Medicine, Kaohsiung Medical University, Kaohsiung 80708, Taiwan; 3 Department of Rehabilitation, Chi Mei Medical Center, Tainan 71004, Taiwan; 4 Department of Occupational Therapy, Chung Shan Medical University, Taichung 40201, Taiwan; dannychi@csmu.edu.tw (W.-C.C.); joelin4700@gmail.com (C.-L.C.); 5 Occupational Therapy Room, Chung Shan Medical University Hospital, Taichung 40201, Taiwan; 6 Occupational Therapy Room, Rehabilitation Department, Taichung Veterans General Hospital, Taichung 40705, Taiwan; s8017550@hotmail.com

**Keywords:** ankle-foot orthosis, standing stability, sit-to-stand, spastic diplegic cerebral palsy

## Abstract

Hinged ankle-foot orthoses (HAFOs) and floor reaction ankle-foot orthoses (FRAFOs) are frequently prescribed to improve gait performance in children with spastic diplegic cerebral palsy (CP). No study has investigated the effects of FRAFO on sit-to-stand (STS) performance nor scrutinized differences between the application of HAFOs and FRAFOs on postural control. This study compared the effects of HAFOs and FRAFOs on standing stability and STS performance in children with spastic diplegic CP. Nine children with spastic diplegic CP participated in this crossover repeated-measures design research. Kinematic and kinetic data were collected during static standing and STS performance using 3-D motion analysis and force plates. Wilcoxon signed ranks test was used to compare the differences in standing stability and STS performance between wearing HAFOs and FRAFOs. The results showed that during static standing, all center of pressure (COP) parameters (maximal anteroposterior/mediolateral displacement, maximal velocity, and sway area) were not significantly different between FRAFOs and HAFOs. During STS, the floor reaction force in the vertical direction was significantly higher with FRAFOs than with HAFOs (*p* = 0.018). There were no significant differences in the range of motion in the trunk, knee, and ankle, the maximal velocity of COP forward displacement, completion time, and the force of hip, knee, and ankle joints between the two orthoses. The results suggest both FRAFOs and HAFOs have a similar effect on standing stability, while FRAFOs may benefit STS performance more compared to HAFOs.

## 1. Introduction

Cerebral palsy (CP) is a neurological disorder caused by a nonprogressive brain lesion or malformation in the child’s developing brain. CP affects primarily motor function and is often accompanied by disturbances of sensation, perception, cognition, communication, behavior, and secondary musculoskeletal problems [1]. Traditionally, individuals with cerebral palsy have been classified by motor type and topographical distribution. Motor types, or the tonal or movement abnormalities, include terms such as spastic, hypotonic, dyskinetic, ataxic, or mixed. The topographic classifications include the limbs that are affected, namely monoplegia, hemiplegia, triplegia, diplegia, or quadriplegia. Spastic diplegic motor disorders are most common in children with CP [2]. Children with spastic diplegic CP suffer from spasticity and have arm involvement of lesser severity than leg involvement. These children with CP experience significant sitting, standing, and walking problems due to spasticity, excessive muscle weakness, kinematic joint abnormalities, and reduced postural control [3,4,5,6,7,8].

For evaluating the functional consequences in children with CP, several classification systems using a simple ordinal grading system of functional capacity have been developed. For the key function of ambulation, the Gross Motor Function Classification System (GMFCS) is the most established and recognized of the functional classification measures in CP. The GMFCS is a simple, five-level, ordinal grading system created to group individuals with CP into one of five levels based on functional mobility or activity limitation. First described in 1997 by Palisano et al. [9], the GMFCS describes the self-initiated movement and use of assistive devices for mobility during an individual’s usual activity. Based on the GMFCS, an individual classified in GMFCS Level I can walk without limitations; Level II can walk with limitations; Level III can often walk with a handheld mobility device indoors; Level IV can use methods of mobility that require physical assistance or powered mobility in most settings; Level V has limited or no walking ability. This classification system was initially designed to be used with children 2–12 years of age. The GMFCS was later expanded and revised (i.e., GMFCS E&R) in 2007 to include ages 12–18, as well as to increase descriptors and differentiation for the levels based on the child’s age [10].

Sitting and standing are prerequisite postural parameters required for performing daily activities, including play and self-care. In addition to more static postures of sitting and standing, the sit-to-stand (STS) movement is a transition movement that is fundamental for upright mobility [11,12,13], and the interaction between children and their environment [14]. As a crucial predictor of functional mobility for children with CP [15], STS movement requires trunk and lower extremity range of motion, muscle strength including knee and hip peak joint moments [16,17,18,19], and synergic muscle activation [20,21], as well as postural reactions [22]. STS movement performance is often impaired in children with CP. To compare the STS movements of typically developing children with CP, Yonetsu et al. [23] reported the characteristics of STS movements in children with spastic CP composed of 13 children in GMFCS I, 8 in GMFCS II, 20 in GMFCS III, and 9 in GMFCS IV. In comparison with typically developing children, some of the CP subjects showed greater trunk forward movement in the first phase, and the other showed little of this movement. The authors assumed that the phenomenon of the large forward trunk was due to immaturity of the equilibrium function, muscular weakness of lower extremities, or had hypertonus in the leg region, while the subjects showed little forward trunk movement had hypertonus in the femoral region. Those who showed little forward trunk movement also moved buttocks forward to shift the center of mass forward. They also reported that some of the CP subjects exhibited extended knee joints before the hip off the seat. A large trunk movement and early abrupt knee extension observed in children with spastic CP concurred with Park et al.’s study [24]. Furthermore, slower speed, decreased knee extensor moment, and decreased maximum power of the hip and knee extensor were also reported in children with CP compared to typically developed peers [24].

The United Cerebral Palsy Association had reported that among an estimated 764,000 people in the United States who have one or more symptoms of CP, almost one-third of children with CP need external support or assistive devices to help them stand up from a sitting position [25]. Various types of orthoses are used to enhance the standing and walking of children with CP [26]. One such intervention involves the use of ankle-foot orthoses (AFOs) [27]. AFO is typically used to improve standing and gait by controlling ankle joints to normalize joint kinetics and joint kinematics and lower walking energy cost to enhance walking efficiency [28,29]. Various AFO types, such as hinged, solid, and floor-reaction AFOs, have been developed for different therapeutic indications [30,31,32,33], however, controversial effects were reported in previous studies.

Dalvand et al. [34] used the Gross Motor Function Measure to compare the effects of hinged AFOs (HAFOs) and solid AFOs (SAFOs) on standing and walking abilities in children with spastic diplegia classified in GMFCS E&R from Level I to Level III. They reported that both SAFO and HAFO improved walking, running, and jumping performance for children with spastic diplegia. Further analysis revealed significantly greater improvement for children wearing HAFOs compared to SAFOs. On the other hand, Rha, Kim, and Park [35] reported that wearing HAFOs did not significantly change the excursion and the speed of postural sway during quiet standing in children with spastic diplegia compared to the barefoot condition. Although the difference was not statistically significant, mediolateral (ML) displacement was noted lower while wearing HAFOs, suggesting that HAFOs might contribute to the enhanced ankle strategy for balance control in ML direction.

To evaluate the effect of AFOs on STS performance, Park et al. [12] used a three-dimensional (3D) motion analysis system to analyze the effect of HAFOs on STS transfer in children with spastic diplegic CP. The results indicated that wearing the HAFO significantly shortened the total duration of STS transfer when compared with that for the barefoot condition. Additionally, the HAFO significantly increased the initial knee flexion, initial and final angle of ankle dorsiflexion, and hip and knee joints’ maximal moment and power. Wilson et al. [36] also used a 3D motion analysis system to compare the effects of HAFOs and SAFOs on the transition from sitting to standing among children with spastic diplegia. They reported that HAFOs improved the time to reach stable standing and ankle dorsiflexion and appeared to be more effective in decreasing the time it takes to reach stable standing compared to SAFOs.

As mentioned, the beneficial effects of HAFOs and SAFOs on standing and STS abilities have been widely studied in the literature. Besides these two types of AFOs, a floor-reaction ankle-foot orthosis (FRAFO) has been developed for children who stand and walk with excessive knee flexion, also known as crouch position, which is a common gait pattern in children with spastic diplegia. FRAFOs can limit ankle dorsiflexion and increase external knee extension by altering the ground reaction force in the sagittal plane. A few studies have examined the benefits of FRAFOs, showing that it could effectively reduce or eliminate the crouch position in children with spastic CP [7,37,38,39,40,41]. However, little is known about the benefits of FRAFOs on postural control mechanisms in static standing in children with CP. Furthermore, evidence demonstrating the effects of FRAFOs on STS performance in these children appears to be lacking. Therefore, the purpose of this study was to compare the effects of hinged and floor-reaction AFOs on standing stability and STS performance in children with spastic diplegic CP. We hypothesized that wearing the HAFOs would improve standing stability compared to wearing FRAFOs as indicated by the measurements of displacement and sway area of the center of pressure (COP). Whereas wearing the FRAFOs would provide upward force and enhance STS performance compared to wearing HAFOs as indicated by the measurements of the ROM of the trunk and lower extremity, and the kinetic data measured by the force plate.

## 2. Materials and Methods

### 2.1. Participants

A crossover repeated-measures design, with participants serving as their own controls, was used for the study. Nine children were recruited from the rehabilitation department of a medical center in central Taiwan. The inclusion criteria were as follows: (1) diagnosed as spastic diplegic CP; (2) age between 5 to 17 years; (3) have worn HAFOs for six weeks; (4) could stand independently for at least 30 s; (5) GMFCS E&R level of II-III; (6) the ability to communicate and follow instructions. The exclusion criteria were: (1) inability to perform study requirements/procedures; (2) surgical intervention three months before the study onset. Participants and their parents provided informed consent for participation. The Institutional Review Board of the Taichung Veterans General Hospital approved this study (authorization number: CF15295A).

### 2.2. Instrumentation

The kinematic data were obtained using a 6-camera motion analysis system (Eagle Digital Realtime system, Motion Analysis Corporation, Rohnert Park, CA, USA). Twenty-two retro-reflective spherical markers were placed on anatomical landmarks according to Helen Hayes marker set: the seventh cervical spine (C7), acromions, sacrum, lateral and medial epicondyles, right and left antero-superior iliac spines, thighs, lateral and medial condyles of the femur, shanks, lateral and medial malleolus, metatarsal heads (between the second and third toes), and calcaneus (heels). Kinematic data were sampled at 60 Hz for static standing and STS movements. Two force plates, each sized 45 cm × 50 cm (Bertec, Columbus, OH, USA), were positioned together and used to collect COP data at a sampling rate of 1200 Hz during standing and STS.

### 2.3. Procedures

Before the experiment, demographic data were obtained through interviews with children’s parents and from their medical charts (i.e., gender, age, type of CP, GMFCS level, and duration of HAFOs usage). Subsequently, passive range of motion (ROM) and muscle tone (using the Modified Ashworth Scale) of the lower extremities were evaluated for each participant. Passive ROM and muscle tone were tested separately as part of a laboratory-based observation. Each participant obtained a pair of customized FRAFOs made from an orthotic manufacturer and was instructed to wear the orthoses for at least four hours per day for six weeks during their daily living activities. The FRAFO is a custom fabricated, molded plastic device made of 3-mm thickness polypropylene. The HAFO blocks ankle plantarflexion but allows free dorsiflexion through the hinge, while the FRAFO has a solid ankle component with no ankle joint and consists of an anterior shell that places the extension force close to the knee.

**Figure 1:**
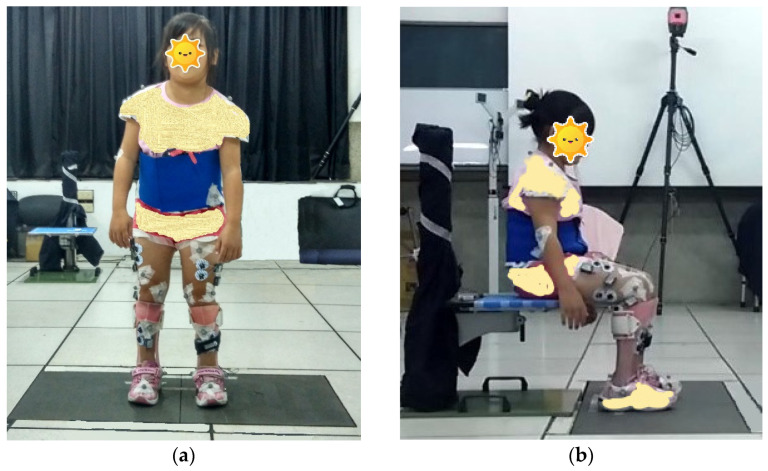
Postural tasks in the study: (**a**) static standing; (**b**) sit-to-stand.

All participants completed the static standing and STS tests with HAFOs at the beginning of this study. Then, after they have worn the FRAFOs for six weeks, the static standing and STS tests were administered again with participants wearing FRAFOs. The standing and STS tasks were practiced for several minutes before their first performance. In static standing, subjects stood with feet shoulder-width apart, one foot on each force plate. They were instructed to keep their head facing forward and stand as still as possible for 30 s with the arms being at their sides (Figure 1a). In STS, each subject’s chair height was adjusted to allow for knee and hip angles of 90° during sitting (Figure 1b). Both feet were kept shoulder-width apart, one foot on each force plate. Participants were instructed to rise from a chair at their comfortable speed without hand(s) supporting their walking aids. Measurements were collected from two valid trials (i.e., the experimental tasks were successfully completed by the participant and the data were also successfully collected), and the mean was obtained for data analysis.

### 2.4. Data Analysis and Statistical Analysis

Biomechanical software Visual 3D (v3.9, C-Motion Inc., Germantown, MD, USA) was used to calculate ankle, knee, and hip joint angles and joint moments based on the attached markers and ground reaction force (GRF). Marker and force plate data were filtered using a zero-phase low-pass 4th order Butterworth filter with a cut-off frequency of 10 Hz [42]. Other related parameters were subsequently obtained using a self-developed program with Matlab software (v2018a, MathWorks Inc., Natick, MA, USA) in the static standing and STS process. In the static standing experiment, the maximal anteroposterior (AP) displacement, maximal ML displacement, maximal velocity, and sway area (i.e., the area which encloses the data points of the trajectory) of COP during mid-20 s were calculated from GRF. In the STS experiment, the ROM of the trunk, knee, and ankle joints, maximal velocity of COP forward displacement, GRF in the vertical direction, completion time, and joint moment of the lower extremity during STS were analyzed. The GRF in the vertical direction was normalized to body weight. The start and end of STS were identified by the signal of GRF, consistent with the study of Zijlstra et al. [43]. The start of standing-up was defined as the first deflection from the baseline of the force platform recording, which was the time when the vertical force was greater than 10% of the baseline. Seat off was defined as the time of the peak GRF, and when the vertical force equaled to the body weight following the peak vertical force, it was counted as the end of standing up.

Wilcoxon signed ranks test was used to compare the differences in standing stability and STS performance between HAFOs and FRAFOs. Differences were considered statistically significant at *p* values < 0.05.

## 3. Results

Nine children (seven males and two females) with spastic diplegic CP participated in this crossover repeated-measures design research. The demographics and gross motor function of the participants are shown in Table 1. The participants had a mean age of 11.11 ± 3.37 years, a mean height of 140 ± 22.28 cm, and a mean weight of 35.44 ± 18.17 kg. Five participants had GMFCS level III and needed a walking aid for walking assistance, whereas four of them had GMFCS level II who could walk and stand up independently. Passive ROMs of joints in both lower limbs, except the ankle joints, were within the normal range for most participants. Six participants had contractures in ankle joints. The Modified Ashworth Scale scores in participants’ lower limb muscles ranged from 0 to 2, except for ankle plantar flexors. The scores of ankle plantar flexors ranged from 3 to 4.

During static standing with HAFOs, all values of COP parameters (maximal AP/ML displacement, maximal velocity, and sway area of COP) were likely to be smaller than wearing FRAFOs. However, the differences were non-significant (*p* > 0.05) (Table 2). During STS, the GRF in the vertical direction was significantly higher with FRAFO than with HAFO (*p* = 0.018). The differences in ROM of the trunk, knee, and ankle, maximal velocity of COP forward displacement, completion time, and the force of hip, knee, and ankle joints for STS were non-significant (*p* > 0.05) between FRAFO and HAFO (Table 3).

## 4. Discussion

In this study, we compared the effects of HAFOs and FRAFOs on standing stability and STS performance in children with spastic diplegic CP. To our knowledge, this is the first study to compare the effects of FRAFOs and HAFOs on STS performance. We hypothesized that wearing HAFOs would improve standing stability better compared to wearing FRAFOs, whereas wearing FRAFOs would enhance STS performance better compared to wearing HAFOs. The results were not entirely consistent with our hypothesis.

During static standing, the COP movements were not significantly different between wearing HAFOs and FRAFOs, indicating similar standing stability for the application of both types of AFOs. Burtner et al. [44] examined the effects of dynamic AFOs (DAFOs) and SAFOs on standing balance of children with spastic CP during the perturbed condition, and they reported that despite similar muscle recruitment patterns between the two types of AFOs, SAFOs inhibited both the use of ankle strategies and activation of the gastrocnemius, and disorganized muscle-response patterns. On the other hand, Buckon et al. reported a detrimental effect of HAFOs on gait for children with spastic CP at GMFCS level II, including increased energy cost and the peak knee extensor moment in early stance, excessive ankle dorsiflexion, and decreased walking velocity [29]. Nevertheless, Radtka et al. [45] reported no differences on variables of gait for children with CP between DAFOs and SAFOs. In our study, we compared the effects of HAFOs and FRAFOs on static standing balance for only 20 s per trial, which was a less challenging postural task for our participants and might be difficult to detect differences between the two types of AFOs.

During STS performance, GRF in the vertical direction was significantly larger when wearing FRAFOs compared to wearing HAFOs. Hennington et al. [16] reported prolonged duration in the extension phase of STS performance for children with CP. Bahar-Özdemir et al. [46] compared STS performance between wearing HAFOs and SAFOs and reported no difference in the strength of rising between the two types of AFO. Although FRAFOs resemble SAFOs and consist of rigid ankle components, FRAFOs integrate an anterior shell that places the extension force close to the knee, which results in significantly larger vertical GRF compared to the application of HAFOs in our study. Bahramizadeh et al. [37] reported that FRAFOs could decrease the flexion angles of knee joints in children with CP in static standing. In our study, although wearing FRAFOs did not significantly increase knee extension angles of biomechanical characteristics of STS kinematics, there was a trend of increased trunk extension angles compared to wearing HAFOs. Therefore, FRAFOs might assist children with spastic diplegic CP to assume an upright posture in the extension phase of STS by providing increased GRF.

In this study, there were no differences between the application of FRAFOs and HAFOs regarding the maximum velocity of COP along the forward direction, and the completion time of biomechanical characteristics of STS. This was partially in agreement with the study results of Bahar-Özdemir et al. [46], in that they reported no differences in terms of weight transfer and sway velocity between SAFOs and HAFOs. Hence, wearing FRAFOs and HAFOs had similar movement efficiency for STS of children with spastic diplegic CP [15,37].

The major limitation of this study was the small sample size. Precautions are warranted when generalizing the study results due to a rather small sample size that might not be representative. It also rendered the results of type II errors with small power that masked significant differences. Nonetheless, a consistent trend that was in line with the findings of previous studies had been observed. Furthermore, according to Rodby-Bousquet and Hagglund [25], children with CP at different functional levels required different amounts of support in STS performance, and the amount of support needed to accomplish STS tasks was also significantly different between subtypes of CP. Since our participants were children with spastic diplegic CP whose functional levels ranged from GMFCS level II to level III, the results may not apply to children with functional levels other than GMFCS II and III or children with CP other than spastic diplegia type. Another limitation of the current study was it focused on biomechanical aspects and lacks for examination of the effects of AFOs on the functional level as well as subjective appraisal of children. Radtka et al. [45] suggested consideration of personal preferences when prescribing AFOs for children with spastic CP. Future studies might explore the fit between specific AFO and functional tasks by evaluating the effects of selective types of AFO across different daily activities and exploring both subjective and objective outcomes at the participation level, this may generate more comprehensive knowledge to inform client-center practice of AFO prescription.

## 5. Conclusions

In summary, the findings of this study indicated that for the nine children with spastic diplegic CP at GMFCS levels II-III, HAFOs and FRAFOs had a similar effect on postural stability; and wearing a FRAFO was beneficial for STS performance that it provided a significantly increased vertical GRF to assist children with spastic diplegic CP to stand up from a sitting position. Since most daily activities are characterized by the frequent postural transition between sitting and standing, therefore, FRAFOs may be more favorable for daily use compared to HAFOs for children with spastic diplegia.

## Figures and Tables

**Table 1:** Demographic characteristics of the participants.

No	Gender	Age (Year)	Height (cm)	Weight (kg)	GMFCS	MAS	WA
1	Male	7	116	19	III	0~3	Yes
2	Male	13	152	40	III	1~2	Yes
3	Female	11	120	31	II	0~3	No
4	Male	17	168	62	III	1+~4	Yes
5	Male	8	122	33	III	0~1+	Yes
6	Male	14	176	68	II	1~1+	No
7	Male	9	130	22	III	0~4	Yes
8	Female	8	125	25	II	0~3	No
9	Male	13	151	19	II	0~3	No
Mean ± SD		11.11 ± 3.37	140 ± 22.28	37.44 ± 18.17			

GMFCS, Gross Motor Function Classification System. MAS, Modified Ashworth Scale. WA, walking aid. The MAS grades ranged from 0 to 4, including 0, 1, 1+, 2, 3, 4; 1+ would be one of the MAS grades.

**Table 2:** Comparison of the center of pressure (COP) parameters during static standing between participants wearing HAFOs and FRAFOs.

COP Parameters	HAFOs		FRAFOs		*p*
	Median	IQR	Median	IQR	
Maximal AP displacement (cm)	4.1	2.85–6.1	6.9	4.55–9.8	0.110
Maximal ML displacement (cm)	5.5	4.15–9.3	8.6	5.55–9.75	0.139
Maximal Velocity (cm/s)	29.59	15.95–42.11	32.21	15.01–43.43	0.214
Sway area (cm^2^)	10.0	5–20	30.0	15–50	0.149

HAFOs, hinged ankle-foot orthoses; FRAFOs, floor reaction ankle-foot orthoses; IQR, interquartile range.

**Table 3:** Comparison of ROM, joint force, maximal FD velocity of COP, GRF in the vertical direction, and completion time for sit-to-stand performance between participants wearing HAFOs and FRAFOs.

Parameters for STS	HAFOs		FRAFOs		*p*
	Median	IQR	Median	IQR	
**Range of motion (degrees)**					
Trunk extension	12.34	7.8–41.6	20.26	11.7–51.2	0.767
Knee extension (R)	26.48	22.1–36.1	28.02	22.4–35.7	0.678
Ankle plantar flexion (R)	3.36	1.5–19.2	2.64	1.9–4.9	0.214
Knee extension (L)	13.73	7.9–20	10.39	9–16.7	0.859
Ankle plantar flexion (L)	2.60	1.7–13	2.24	1.9–4.1	0.314
**Joint force (Nm/kg)**					
Hip extension (R)	1.05	0.32–1.78	0.65	0.35–2.05	0.515
Knee extension (R)	0.77	0.67–1.51	0.92	0.31–1.83	0.859
Ankle plantar flexion (R)	0.15	0.1–0.58	0.18	0.09–0.83	0.139
Hip extension (L)	0.67	0.5–0.83	0.57	0.51–1.27	0.594
Knee extension (L)	0.57	0.51–0.86	0.70	0.43–0.87	0.515
Ankle plantar flexion (L)	0.13	0.08–0.25	0.18	0.1–0.24	0.678
**Maximal FD velocity of COP (cm/s)**	233.0	131.02–389.41	274.01	108.03–393.88	0.594
**GRF in the** **vertical** **direction (N/kg)**	1.17	0.93–1.3	1.28	1.05–1.33	0.018 *
**Completion time (s)**	3.57	3.12–5.05	3.59	3.1–5.47	0.678

HAFOs, hinged ankle-foot orthoses; FRAFOs, floor reaction ankle-foot orthoses; IQR, interquartile range; R, right; L, left; FD, forward displacement; COP, the center of pressure; GRF, ground reaction force. * *p* < 0.05.

## Data Availability

The data that support the findings of this study are available from the corresponding author upon reasonable request due to ethical and privacy restrictions.
